# Supplementary material for: Parasagittal dural volume correlates with cerebrospinal fluid volume and developmental delay in children with autism spectrum disorder
Source: Commun Med (Lond). 2024 Oct 4;4:191. doi: 10.1038/s43856-024-00622-8 (PMC11452566; doi:10.1038/s43856-024-00622-8)
Supplement: Supplementary file 2 — Supplementary material [file 43856_2024_622_MOESM2_ESM.pdf]

Supplementary Materials

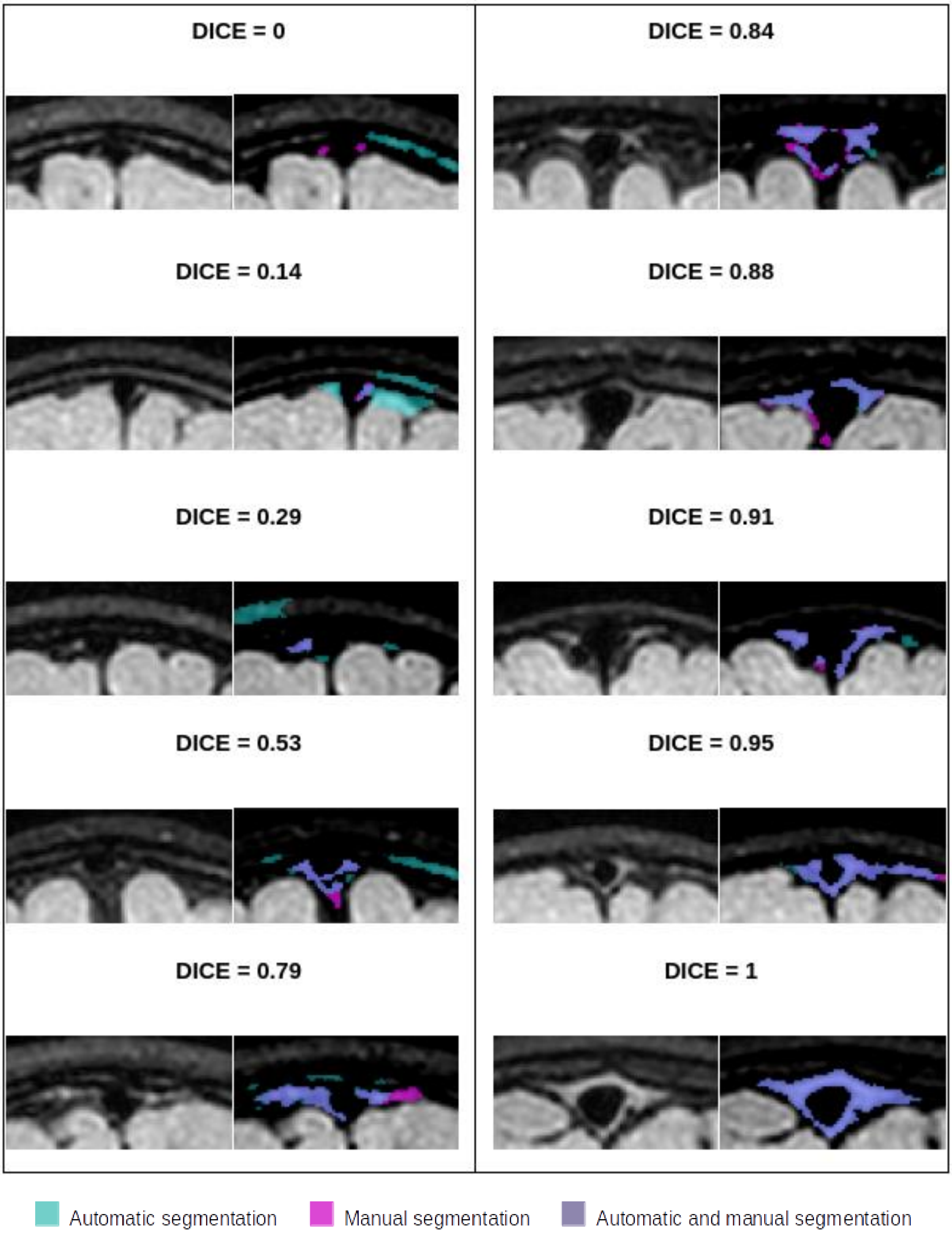

Fig. S1: The above images illustrate automatic segmentation masks of PSD overlapped with manually corrected masks from ten randomly selected images from the entire cohort. Each example shows cropped FLAIR images on the left side, while on the right side, FLAIR images are displayed overlapped with both manual and automatic segmentations. Additionally, the DICE-score corresponding to each example is presented.

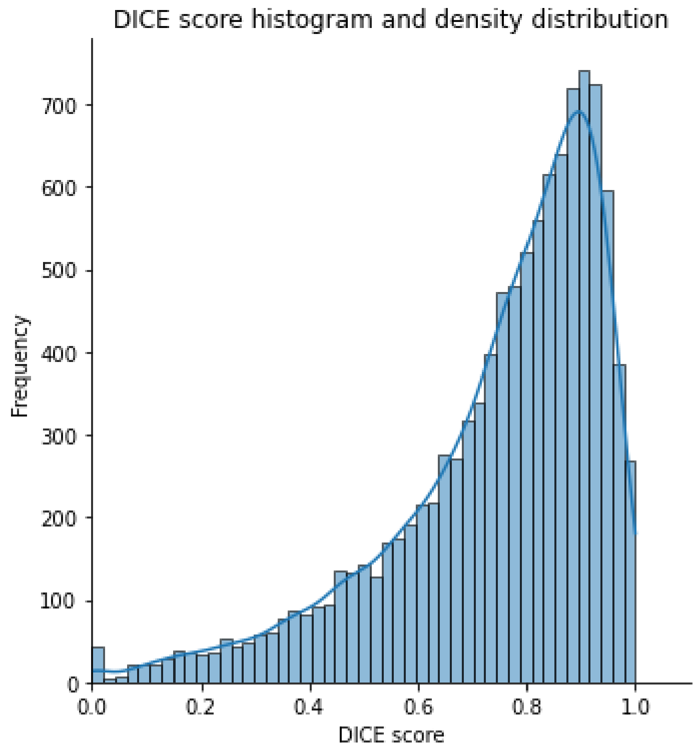

Fig. S2: The graph illustrates the histogram and density distribution of DICE-scores, comparing the manually corrected segmentation of PSD as the ground truth with automatic segmentation of PSD across 56 children with ASD.
