# Supplementary material for: Parasagittal dural volume correlates with cerebrospinal fluid volume and developmental delay in children with autism spectrum disorder
Source: Commun Med (Lond). 2024 Oct 4;4:191. doi: 10.1038/s43856-024-00622-8 (PMC11452566; doi:10.1038/s43856-024-00622-8)
Supplement: Supplementary file 3 — Reporting Summary [file 43856_2024_622_MOESM3_ESM.pdf]

nature portfolio

Corresponding author(s): DBPR: COMMSMED-23-0841-T

Last updated by author(s): Jul 10, 2024

Reporting Summary

Nature Portfolio wishes to improve the reproducibility of the work that we publish. This form provides structure for consistency and transparency in reporting. For further information on Nature Portfolio policies, see our Editorial Policies and the Editorial Policy Checklist.

Statistics

For all statistical analyses, confirm that the following items are present in the figure legend, table legend, main text, or Methods section.

n/a	Confirmed	
☐	☒	The exact sample size (*n*) for each experimental group/condition, given as a discrete number and unit of measurement
☐	☒	A statement on whether measurements were taken from distinct samples or whether the same sample was measured repeatedly
☐	☒	The statistical test(s) used AND whether they are one- or two-sided<br>*Only common tests should be described solely by name; describe more complex techniques in the Methods section.*
☐	☒	A description of all covariates tested
☐	☒	A description of any assumptions or corrections, such as tests of normality and adjustment for multiple comparisons
☐	☒	A full description of the statistical parameters including central tendency (e.g. means) or other basic estimates (e.g. regression coefficient) AND variation (e.g. standard deviation) or associated estimates of uncertainty (e.g. confidence intervals)
☐	☒	For null hypothesis testing, the test statistic (e.g. *F*, *t*, *r*) with confidence intervals, effect sizes, degrees of freedom and *P* value noted<br>*Give P values as exact values whenever suitable.*
☒	☐	For Bayesian analysis, information on the choice of priors and Markov chain Monte Carlo settings
☒	☐	For hierarchical and complex designs, identification of the appropriate level for tests and full reporting of outcomes
☐	☒	Estimates of effect sizes (e.g. Cohen's *d*, Pearson's *r*), indicating how they were calculated

Our web collection on statistics for biologists contains articles on many of the points above.

Software and code

Policy information about availability of computer code

Data collection	The MRI data was collected from the hospital picture archiving and communication system (PACS); based on dcm4che open-source database.
Data analysis	Image analysis: To extract PSD volumes, 3D T2w FLAIR images were used. The steps involved were as follows: a) conversion of FLAIR and T1 images from dicom to niftii format images using DCM2NII software version no. v1.0.20170724; b) Preprocessing was performed using FSL: 5.0; ROBEX: v12; ANTS: 31/07/2017; MATLAB: R2020a; c) a neuroradiologist identified the anterior commissure (AC) and posterior commissure (PC) of the corpus callosum and a 60 degree angle was define PSD segment of interest. d) The 10 manual segmentations were used to train a 2D UNET algorithm. e) AI-derived segmentations were reviewed and a manual correction was performed on each slice for each subject using FSLeyes v5.0. f) the trained neural network was used to segment the PSD of the 56 participants and manual correction was performed for each slice and for each subject to improve accuracy by the same person. g) gray matter, white matter, intracranial and CSF volumes were calculated using ANTS cortical thickness. Extra-axial CSF volume was derived from CSF mask by manually removing the ventricles and CSF space below the AC-PC line.<br><br>Statistical analyses were performed using software R: 4.3.1. Correlations were determined by Kendall correlation coefficient. Statistical significance was accepted at the 0.05 level (two-tailed).

For manuscripts utilizing custom algorithms or software that are central to the research but not yet described in published literature, software must be made available to editors and reviewers. We strongly encourage code deposition in a community repository (e.g. GitHub). See the Nature Portfolio guidelines for submitting code & software for further information.

Data

Policy information about availability of data

All manuscripts must include a data availability statement. This statement should provide the following information, where applicable:

- Accession codes, unique identifiers, or web links for publicly available datasets
- A description of any restrictions on data availability
- For clinical datasets or third party data, please ensure that the statement adheres to our policy

Data is available from the corresponding author on reasonable request. Code and software versions used can be found at https://doi.org/10.6084/m9.figshare.24582369.v4

Research involving human participants, their data, or biological material

Policy information about studies with human participants or human data. See also policy information about sex, gender (identity/presentation), and sexual orientation and race, ethnicity and racism.

Reporting on sex and gender	The training dataset analysis was performed on three self-reporting males and seven females. Of the 56 subjects in the patient cohort who met our inclusion criteria, only 8 were of female sex, based on clinical records.
Reporting on race, ethnicity, or other socially relevant groupings	Not relevant
Population characteristics	48 patients of male sex and 8 patients of female sex with a diagnosis of autism based on Autism Diagnositic Interview - Revised (ADI-R) and Autism Diagnostic Observation Schedule - second edition (ADOS-2) criteria were included. The degree of developmental delay was evaluated using either the Wechsler Intelligence Scale for Children (WISC-IV) or the Wechsler Preschool and Primary Scale of Intelligence - III (WPPSI-III). Age: 4.43 ± 1.51 Healthy adults (3m;7f, age 32.6 ± 12.5 years) without neurological or psychiatric disorder.
Recruitment	Healthy adults were recruited by word of mouth. All patients discharged from the Child Neuropsychology Unit at our Institute, with a clinical diagnosis of autism and who also underwent an MRI from Jan 2022 to March 2023 were recruited.
Ethics oversight	Scientific Insitute IRCCS Eugenio Medea Ethical board approval protocol no. 1022

Note that full information on the approval of the study protocol must also be provided in the manuscript.

Field-specific reporting

Please select the one below that is the best fit for your research. If you are not sure, read the appropriate sections before making your selection.

☒ Life sciences ☐ Behavioural & social sciences ☐ Ecological, evolutionary & environmental sciences

For a reference copy of the document with all sections, see nature.com/documents/nr-reporting-summary-flat.pdf

Life sciences study design

All studies must disclose on these points even when the disclosure is negative.

Sample size	No sample-size calculations could be performed. There is no information in the literature regarding PSD in the developing brain. Therefore making any predictions is impossible at this point in time.
Data exclusions	We analysed the age distribution curve of the intial cohort and identified a limited number of participants below the age of 2 years and above the age of 8 years. In order to minimize variability, we opted to exclude the extreme ends of the age distribution curve. Additionally, two patients were excluded due to inadequate FLAIR image quality.
Replication	This is a single-site study, wherein all images were acquired on the same MRI scanner using identical imaging parameters. The entire process, from image acquisition to generating output results, followed a uniform methodology, including the use of identical software versions. To enhance the accuracy of AI-derived PSD volumes, manual corrections were implemented by a sole individual. Such analysis have not been performed thus far in the developing human brain.
Randomization	No randomisation was required in this observational study.
Blinding	The clinical diagnosis of autism was known to all investigators. However, investigators running AI and manual segmentation protocol were not aware of clinical severity scales.

Reporting for specific materials, systems and methods

We require information from authors about some types of materials, experimental systems and methods used in many studies. Here, indicate whether each material, system or method listed is relevant to your study. If you are not sure if a list item applies to your research, read the appropriate section before selecting a response.

Materials & experimental systems

n/a	Involved in the study
☒	☐ Antibodies
☒	☐ Eukaryotic cell lines
☒	☐ Palaeontology and archaeology
☒	☐ Animals and other organisms
☐	☒ Clinical data
☒	☐ Dual use research of concern
☒	☐ Plants

Methods

n/a	Involved in the study
☒	☐ ChIP-seq
☒	☐ Flow cytometry
☐	☒ MRI-based neuroimaging

Clinical data

Policy information about clinical studies
All manuscripts should comply with the ICMJE guidelines for publication of clinical research and a completed CONSORT checklist must be included with all submissions.

Clinical trial registration	This study is registered under ClinicalTrials.gov
Study protocol	This study is registered with the Scientific Institute IRCCS Eugenio Medea Hospital Registry, Protocol no. 1022
Data collection	The time frame for patient recruitment was from January 2022 to March 2023. FLAIR and T1 weighted images were downloaded from the hospital PACS system.
Outcomes	Primary outcome: 2D-UNET derived PSD volume obtained from FLAIR images; Secondary outcome: total and extra-axial cerebrospinal fluid, gray matter, white matter and intracranial volume. Clinical measures that define severity of autism and developmental delay using validated scales such as ADOS-2, ADI-R and GDS.

Plants

Seed stocks	*Report on the source of all seed stocks or other plant material used. If applicable, state the seed stock centre and catalogue number. If plant specimens were collected from the field, describe the collection location, date and sampling procedures.*
Novel plant genotypes	*Describe the methods by which all novel plant genotypes were produced. This includes those generated by transgenic approaches, gene editing, chemical/radiation-based mutagenesis and hybridization. For transgenic lines, describe the transformation method, the number of independent lines analyzed and the generation upon which experiments were performed. For gene-edited lines, describe the editor used, the endogenous sequence targeted for editing, the targeting guide RNA sequence (if applicable) and how the editor was applied.*
Authentication	*Describe any authentication procedures for each seed stock used or novel genotype generated. Describe any experiments used to assess the effect of a mutation and, where applicable, how potential secondary effects (e.g. second site T-DNA insertions, mosiacism, off-target gene editing) were examined.*

Magnetic resonance imaging

Experimental design

Design type	Structural MRI. No functional MRI was performed
Design specifications	No functional MRI data to specify
Behavioral performance measures	No functional MRI data to specify

Acquisition

Imaging type(s)	Structural
Field strength	3T
Sequence & imaging parameters	Both sequences used in this manuscript were acquired in the sagittal plane with isotropic 1x1x1mm3 voxel resolution.

Sequence & imaging parameters

3D-T1 weighted (3D-T1w): repetition time (TR) = 8,3 ms; echo time (TE) = 3,9 ms; echo train length (ETL) = 256; flip angle = 8°; 1 average; Acquisition time: 5 min and 38 s. 3D T2-Fluid Attenuated Inversion Recovery (3D-FLAIR): TR = 4800 ms; TE = 298 ms; inversion time = 1650 ms; ETL = 167; flip angle = 90°; 2 averages; 1x1x1 mm3 voxel size. Acquisition time: 6 min.

Area of acquisition

Brain

Diffusion MRI ☐ Used ☒ Not used

Preprocessing

Preprocessing software

Preprocessing was performed on both T1w and FLAIR images. In particular, T1w preprocessing included: image orientation according to a standard orientation, intensity scaling to an average of value (100), rigidly moving the image to the MNI space, run ANTs cortical thickness and fsl_anat on the bias field corrected image. FLAIR preprocessing instead, included only N4 Bias Field correction and rigid registration to the T1w3D image.
All these steps were performed using FSL(5.0), ANTs (31/07/2017) and ROBEX(v12)

Normalization

T1w images intensity scaled to an average of value (100)

Normalization template

none used

Noise and artifact removal

none used

Volume censoring

not performed

Statistical modeling & inference

Model type and settings

this was an anatomical study exploring the volume of PSD, white matter, gray matter, intracranial volume and cerebrospinal fluid in the developing brain

Effect(s) tested

ANOVA test was used for assessing differences between groups in categorical variables.

Specify type of analysis: ☒ Whole brain ☐ ROI-based ☐ Both

Statistic type for inference

(See Eklund et al. 2016)

Not relevant

Correction

Not relevant

Models & analysis

n/a	Involved in the study
☒	☐ Functional and/or effective connectivity
☒	☐ Graph analysis
☒	☐ Multivariate modeling or predictive analysis
